# Protection against Bovine Respiratory Syncytial Virus Afforded by Maternal Antibodies from Cows Immunized with an Inactivated Vaccine

**DOI:** 10.3390/vaccines11010141

**Published:** 2023-01-09

**Authors:** Gilles Meyer, Charlotte Foret-Lucas, Maxence Delverdier, Antoine Cuquemelle, Aurélie Secula, Hervé Cassard

**Affiliations:** Interactions Hôtes-Agents Pathogènes (IHAP), Université de Toulouse, INRAE (Institut National de Recherche pour L’agriculture, L’alimentation et L’environnement), ENVT (Ecole Nationale Vétérinaire de Toulouse), 31100 Toulouse, France

**Keywords:** BRSV, vaccination, cattle, colostrum, maternal immunity, BRD

## Abstract

The passive protection afforded by the colostrum from cattle that were vaccinated prepartum with an inactivated combination vaccine against the bovine respiratory syncytial virus (BRSV) was evaluated after an experimental challenge of calves. Pregnant cows without or with a low ELISA and neutralizing BRSV antibody titers were twice vaccinated or not vaccinated, the last immunization being at one month prior to calving. Vaccination was followed by a rapid increase in BRSV antibody titers after the second immunization. Twenty-eightnewborn calves were fed during the 6 h following birth, with 4 L of colostrum sourced from vaccinated cows (14 vaccine calves) or non-vaccinated cows (14 control calves) and were challenged with BRSV at 21 days of age. We showed that maternal immunity to BRSV provides a significant reduction in the clinical signs of BRSV in calves, especially for severe clinical forms. This protection was correlated with reduced BRSV detection in the lower respiratory tract but not in nasal swabs, indicating an absence of protection against BRSV nasal excretion. Finally, transcriptomic assays in bronchoalveolar lavages showed no statistical differences between groups for chemokine and cytokine mRNA transcriptions, with the exception of the overexpression of *IL-9* at days 6 and 10 post-challenge, and a severe downregulation of *CXCL-1* at day 3 post-challenge, in the vaccine group.

## 1. Introduction

Bovine respiratory syncytial virus (BRSV), recently referred to as bovine orthopneumovirus [1], is an enveloped, non-segmented, negative-stranded RNA virus that belongs to the Orthopneumovirus genus, within the Pneumoviridae family. This virus is often involved in bovine respiratory disease (BRD) outbreaks, alone or in combination with other respiratory pathogens [2]. As BRD accounts for considerable economical losses and reduction in cattle welfare [3,4], prevention is currently used that is based on biosecurity, husbandry management, and/or vaccination. Vaccination against respiratory viruses, including BRSV, bovine viral diarrhea (BVDV), bovine parainfluenza 3 virus (BPI3), bovine coronavirus (BCoV), and, in some contexts, bovine herpesvirus 1 (BoHV-1), is indeed considered a key management strategy to minimize the mortality and economic losses associated with BRD in young calves [5,6,7,8]. Since BRD occurs most often in the first weeks of life, calves are, in general, vaccinated at a very young age while the maternally derived antibodies are still present. Extensive investigation into the BRSV vaccination of calves in the presence of maternal antibodies has been performed [5,9,10,11,12,13,14,15,16,17,18,19]. Often, the vaccination of calves in the face of maternal antibodies does not result in seroconversion because maternally derived immunity interferes with the activation of adequate antibody responses to vaccination. On the other hand, maternal antibodies do not suppress the priming of the humoral and cellular immune system after vaccination, as indicated by rapid systemic and mucosal IgA responses after a challenge or secondary infection [11,13,14,15,17,18,19,20]. Several factors, including age, level of maternal immunity, type of vaccine, and route of administration (for review, see [5,8]) may affect the outcome of vaccination, potentially resulting in a lack of clinical protection and/or increased risk of virus shedding [5,10,12].

An alternative approach to protecting the calf early in life is the vaccination of the pregnant dam to achieve higher and more homogenous levels of antibodies in the colostrum and, consequently, in calves [21,22]. Epidemiological or experimentally induced infection studies have shown that although maternal antibodies do not prevent BRSV infection, they do reduce the signs of acute clinical disease during the first months of life [5,6,7,8,21,22,23,24,25]. Conversely, there are only a few experiments investigating the efficacy of vaccinating dams to protect calves from BRSV via maternal antibodies in the first weeks of life. One study showed that colostrum from vaccinated dams, as a source of passive immunity, strongly reduced the severity of the clinical signs and the gross and microscopic lung lesions when calves were challenged with BRSV very early, on days 3–6 of life [24]. The objective of this study was to evaluate the passive protection afforded by colostrum from cattle that were vaccinated prepartum with an inactivated combination vaccine against BRSV, bovine parainfluenza type 3 virus (BPI3), and *Mannheimia haemolytica*, employed against a BRSV challenge in calves at 3 weeks of age.

## 2. Materials and Methods

### 2.1. Virus and Inoculum

BRSV-3761 was isolated from the nasal swab (NS) of a calf with BRD. The BRSV-3761 inoculum was produced by five passages in Bovine Turbinate cells (ATCC, CRL 1390), followed by two supplementary passages in newborn calves, as previously described [26]. The inoculum consisted of bronchoalveolar lavage (BAL) of the second passage; it was tested as being free of *Mannheimia haemolytica*, *Pasteurella multocida*, *Histophilus somni*, *Mycoplasma bovis*, BVDV, BPI3, BCoV, and BoHV-1 (Life Technologies, Carlsbad, CA, USA, Lsi Taqvet qPCR, respiratory pathogens, BVDV, and IBR kits, Lissieu, France). The titer of the inoculum was 10^4^ PFU/mL before the challenge and was then 6 × 10^3^ PFU/mL when tested 6 h after experimental infection.

### 2.2. Experimental Design

The animals were housed in biocontainment facilities (A2 level of biosafety), and the experiment was designed in conformance with the guidelines of the European Community Council on Animal Care (2010/63/EU) and under the authority of a license issued by the National Ethics Committee (ref 01735.02, UMR agreement C3155527, French Ministry of Agriculture, Ethics Committee no. 115).

#### 2.2.1. Vaccination of Cows and Colostrum Collection

Thirty-two cows (between their second and fourth gestations) from the same BVDV-free dairy herd (INRA Domaine du Pin experimental station) and non-vaccinated against BRSV were tested by an ELISA (Life Technologies, Carlsbad, CA, USA—LsiVRSB ELISA kit) and serum neutralization (SN) in order to evaluate their initial BRSV-serological status. The indirect ELISA allowed for a qualitative classification of the animals as negative (-), weakly (+), moderately (++), highly (+++), and very highly (++++) positive. They were further divided into two groups, each group containing 6 negative, 6 weakly positive (+), and 4 moderately positive (++) cows. The BRSV antibody status was confirmed by SN just before the first injection of the vaccine, with titers ranging between 0 and 28 ED_50_/mL (50% effective dose) in both groups, according to the Spearman–Kärber titration.

In the first group, cows were given two doses of an inactivated multivalent vaccine directed against BRSV, BPI3, and *M. haemolytica* (nationally licensed trade name: Bovilis^®^ Bovigrip, batch number A075A01 (02762002), MSD Animal Health, Rahway, NJ, USA), spaced one month apart, following the manufacturer’s recommendations (5 mL via subcutaneous injection in the neck). The vaccine administration was timed in such a way that the second injection of the vaccine was given one month (36–27 days) prior to the expected calving. The cows in the second group were not vaccinated.

The serological status of the cows was followed using an ELISA and SN from the sera collected at each vaccination date and just before calving. The absence of natural BRSV infection was checked by the absence of seroconversion of non-vaccinated cows and by the absence of direct BRSV detection by RT-qPCR in nasal swabs, taken each week from the day of the first vaccine injection until calving. At calving, colostrum from the first and second milkings (between 4 and 10 L per cow) were collected and conserved at −20 °C. The antibody status of each colostrum sample was assessed via an ELISA and SN.

#### 2.2.2. Infection of Calves 

**Figure 1:**
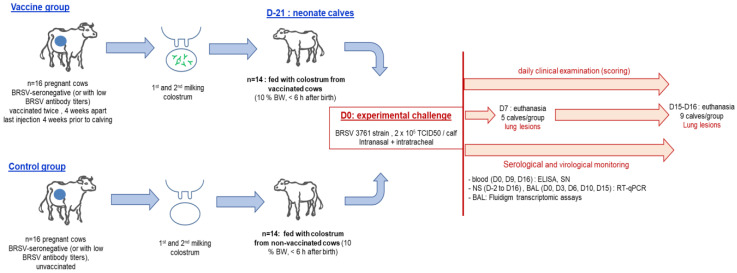
Experiment timeline of the cow vaccinations, calving, and BRSV challenge with sampling. NS: nasal swabs; BAL: broncho-alveolar lavages; SN: seroneutralization; ELISA: enzyme-linked immunosorbent assay.

Twenty-eight male calves (Prim Holstein or Normande breeds) were obtained from the same herd (INRA Domaine du Pin experimental station) three months later. At calving, the calves were immediately separated from their mothers and divided into two groups of 14 calves (Figure 1). Calves in the first group were fed with colostrum from vaccinated cows (immune group), while calves in the second group were fed with colostrum from unvaccinated cows (non-immune group). The colostrum was not pooled, each calf receiving the colostrum from a specific cow (10% body weight within 6 h post-calving). Two colostrum samples per group with the highest SN titers were excluded; finally, 28 calves were used for the challenge.

After the first colostrum meal, the calves were fed with milk replacements. To evaluate the passive transfer of immunity and the kinetics of BRSV antibodies, calves were tested for BRSV serum IgG and SN antibodies before colostrum ingestion, 2 days after birth, each week, and immediately before the challenge. BRSV RT-qPCR was performed from NS each week before the challenge, to rule out natural infection during rearing. The absence of BVDV in calves was assessed at birth via negative RT-qPCR. All calves remained negative for the BPI3 virus during the experiment.

Calves were challenged at 22 ± 3.5 days via intranasal and intratracheal routes of inoculation with the BRSV-3761 inoculum, at a dose of 2 × 10^5^ TCID_50_ per animal (5 and 15 mL by intranasal and intratracheal routes, respectively). At day 7 (D7) post-challenge, five calves in each group were euthanized using an overdose of general anesthesia (5 mg/kg ketamine, followed by 15 mg/kg pentobarbital sodium) for post-mortem examination of the lungs (gross lesions, histopathology, and virus quantification). The remaining 18 calves were euthanized at the end of the experiment at D15–D16.

### 2.3. Clinical Evaluation

A clinical evaluation was performed by the same investigator in a non-blinded way, at the same time twice a day, from 3 days before infection to 16 days after infection (D16), recording body temperature, nasal discharge, coughing, appetite, general state, breathing, respiratory rate (RR), and lung sounds, as already described [26], with slight modifications. Briefly, the scores for RR were 0 (<35 RR/min), 1 (35 < RR < 45), 2 (45 < RR < 60), 3 (60 < RR < 80) and 4 (RR > 80). A score between 0 (normal), 1 (mild), and 2 (severe) was attributed for nasal discharge, coughing, appetite, general state, dyspnea, and lung sound parameters, respectively. For daily clinical scores (CS), a coefficient of 3, 1, 3, 2, 2, 3, and 3 was subsequently attributed for RR, nasal discharge, coughing, decreased appetite, general state, dyspnea, and abnormal lung sound parameters, respectively. The accumulated clinical scores (ACS) of both groups were calculated as the mean of the individual area under daily clinical scores, using the trapezoid method.

In addition, cumulative clinical scores (CCS) were calculated for each calf (the sum of the daily clinical score for each calf, without the use of coefficients) and calves were grouped in five intervals (0–10, 10–30, 30–70, 70–100, >100) according to their CCS. The interval 0–10 corresponds to no or very minor respiratory signs, which are never identified in field conditions. The other ranges correspond to mild (10–30: 35 < RR < 45, infrequent cough), moderate (30–70: 45 < RR < 60, frequent cough), and severe (70–100: RR > 60, constant cough, abdominal dyspnea, loss of appetite, and general state impairment) clinical forms. A score of >100 indicates calves with severe acute respiratory distress syndrome.

Gross lesions were evaluated during necropsy after euthanasia at D7 (5 calves per group) and D15–D16 (end of the experiment). The lungs were examined and photographed, then assessed by palpation of each of the pulmonary lobes to determine the percentage of lung consolidation. Observations were recorded on a standard lung diagram and expressed as the percentage of pneumonic consolidation of the cranial lobes [26]. Tissue samples were collected for histopathological and virologic examinations, including the cranial right and left lobes and the intermediate and caudal left and right lobes of the lungs, as well as the mediastinal and tracheobronchial lymph nodes. For histopathology, tissue samples were paraffin wax-embedded, after fixation in 10% neutral formalin, sectioned at 3–5 µm, and stained with hemalun and eosin. A qualified pathologist described and scored the severity of the histopathology and inflammation on each slide as either normal, minimal, light, moderate, marked, or severe (scored from 0 to 5). A mean score of histopathological severity was calculated for each calf, based on all the lung tissue slides for that animal.

### 2.4. Virological Examination

BRSV detection was performed on the nasal secretions and bronchoalveolar lavages (BAL) using a real-time RT-qPCR assay (Taqvet BRSV/bPI3 Lsi, Lissieu, Life Technologies), according to the manufacturer’s instructions in a Light Cycler 480 (Hoffmann-La Roche Ltd., Bâle, Switzerland). A standard plasmid curve for quantification was obtained by successive ten-fold dilutions of a plasmid containing 10^6^ DNA copies of the same BRSV amplified nucleotide sequence (Life Technologies, Carlsbad, CA, USA).

Nasal swabs were collected from each animal every two days, from D-2 to D16, in 1mL of RLT buffer (Qiagen S.A., Courtaboeuf, France) for real-time RT-qPCR. To follow the BRSV amplification in the lower respiratory tract, BAL was performed via endoscopy, as previously described [27], using 100 mL sterile NaCl supplemented with antibiotics at D0, D3, D6, D10, and D15 for 5 calves of each group. Virus detection was also performed at D7 and D15 in the collected respiratory tissue samples during necropsy (cranial right and left lobes, the intermediate lobes of the lungs, and the mediastinal and tracheobronchial lymph nodes).

In addition, individual accumulated virological shedding (AVS) was calculated for each calf as the area under the BRSV titers, using the trapezoid method.

### 2.5. Evaluation of the Host Response

#### 2.5.1. Humoral Response

Antibody response was checked at D0, D3, D6, D9, D12, and D16 for the detection of BRSV-specific IgG (ELISA LSIVet™ Bovine RSV-Serum, Lsi, Lissieu, Life Technologies, France) and neutralization, as already described [26]. Serum-neutralizing (SN) antibody titers were expressed as 50% of the effective dose (ED_50_), calculated using the Spearman–Kärber method.

Briefly, for ELISA, the corrected optical density or OD (ODc) of each sample was calculated as follows: ODc = AgV OD − AgC OD, where AgV and AgC were the wells with the BRSV viral antigen (AgV, odd columns) and control antigen (AgC, even columns). Then, the E/P (sample/positive) ratio was calculated for each sample, as follows: E/P = ODc Sample/ODc m PC (mean ODc of positive controls). After validation (ODc mPC > 0.6 and ODc mNC (mean ODc of negative controls) < 0.150), the semi-quantitative interpretation was performed, as follows: E/P < 0.1= negative; 0.1 < E/P < 0.2 = doubtful; 0.2 < E/P < 0.4 = positive +; 0.4 < E/P < 0.6 = positive ++; 0.6 < E/P < 0.8 = positive +++; E/P > 0.8 = positive ++++)

#### 2.5.2. Transcriptomic Response in BAL

The BALs at D0, D3, D6, D10, and D15 were analyzed for the calf transcriptomic response of 42 bovine genes involved in the inflammatory response and innate immune response, using the high-throughput microfluidic qPCR platform, BioMark (Fluidigm, South San Francisco, CA, USA), as previously described [28]. Genes and primers are listed in supplementary File S1 in the Supplementary Materials. Once the quality test was passed, the data of the run were exported to the Biogazelle qBase+ software program (Technologiepark-Zwijnaarde, Gent, Belgium; www.biogazelle.com, accessed on 5 December 2022). Relative expressions for each day were calculated via ΔΔCT analysis after normalization (GeNorm analysis) on the two most stable bovine housekeeping genes (*hprt* and *sdha*) from a list of 6 genes previously mentioned in the literature (*gapdh*, *hprt*, *rpl19*, *rpl26*, *sdha*, and *ywha7* genes). The results were expressed as log-transformed calibrated normalized relative quantity (CNRQ) values (Biogazelle qBase + software). For each day and each molecule, fold changes were expressed as the mean CNRQ values of immune calves on the mean CNRQ values of the non-immunel calves.

### 2.6. Statistical Analysis

Statistical analyses for the clinical and virological examinations were performed using GraphPad (La Jolla, CA, USA). Logarithmic transformation was applied to fulfill the conditions of variances in homogeneity and normality when necessary (qPCR data). Data were expressed as the arithmetic mean ± standard error of the mean (SEM) or standard deviations (SD). A two-way ANOVA with repeated measures (a three-factor split-plot ANOVA) was used to analyze the clinical and qPCR results. When the effects of the “day” and “treatment” factors were significant among the interactions, a Bonferroni test between contrasts was used to compare the treatments on each day post-challenge. The *p*-values are indicated in the text; levels of significance are indicated on the graphs with stars: * *p* < 0.05, ** *p* < 0.01, and *** *p* < 0.001. A one-way ANOVA was used to compare the AVS and ACS. When the effect of the “treatment” factor was significant, a Newman–Keuls test was used to compare the treatment effects at each time point. A *t*-test (Mann–Whitney U test) was also run for these parameters.

Statistical analysis of the Fluidigm transcriptomic results was carried out on the log-transformed CNRQ values of mRNA expression, by comparing the slopes from D0 to Dx between the infected and control calves [28]. We used a linear mixed model with a random effect for *group*, considering the interactions between time and status (infected or control) and fit by maximum likelihood *t*-tests, using Satterthwaite approximations to the degrees of freedom (formula: Y ~ Status * Time + (1 | Calves)). This model takes into account the heterogeneity of cytokine RNA data at D0, when the calves were not infected, and the different predictions of the evolution between infected and control groups when the interaction (ANOVA type III) is significant.

## 3. Results

### 3.1. Vaccination Induces a Strong BRSV Antibody Response in Cows and a High BRSV Antibody Titers in Colostrum and Colostrum-Fed Calves

The vaccination of cows was followed by an increase in ELISA antibodies after the first immunization (Table 1). One month after the second vaccine injection (calving date), all the vaccinated cows had ELISA titers that were between high (+++) and very high (++++) (according to the manufacturer’s score calculation), except for one animal that was highly positive at the date of the second vaccine injection but was moderately positive (++) one month later. When testing antibody status via SN, the results indicated only a low seroconversion of neutralizing antibodies after the first vaccine injection, with titers ranging from 0 to 6 Log_2_ EID_50_/mL. After the second vaccine injection, all vaccinated cows were seroconverted, with titers ranging between 7 and 9.2 Log_2_ EID_50_/mL at the date of calving (Table 1). All the non-vaccinated cows remained at their initial ELISA and SN antibody status until calving (Table 1).

As seen in comparisons of the blood titers in the cows, ELISA results were lower in the colostrum as only the colostrum samples of eight vaccinated cows were very highly positive (++++, Table 1). However, these results must be interpreted cautiously because the differences were likely due to the analytical method used, given that (i) the ELISA results were not truly quantitative results, (ii) the matrix was different, and (iii) the ELISA kit was not calibrated or commercialized for colostrum samples. This tendency was also found in the non-vaccinated group, with 12 of the 16 cows having colostrum showing as negative for the BRSV ELISA antibodies. In contrast, the SN results in the vaccinated group showed higher relative titers in the colostrum than in the serum of the mother when expressed in 1 mL samples (Table 1). All colostrum samples from the 16 vaccinated cows were positive, while five colostrum samples of the non-vaccinated cows were positive, with lower titers (Table 1). The four colostrum samples (two per group) with the highest SN titers were excluded and were not distributed to the calves.

The efficacy of the colostrum transfer to the 28 calves was assessed by serological investigation two days after calving and at D0. The ELISA status of all the calves (immune group) that received colostrum from vaccinated cows was between moderately (++) and highly (+++) positive (Table 1) at two days after calving; their status remained constant until the challenge, twenty-one days later. In calves fed with the colostrum of non-vaccinated cows (non-immune group), the ELISA status was negative for 11 calves and weakly positive (+) for 3 calves at one day after calving (Table 1). The SN antibody titers for BRSV two days after calving ranged between 7.2 and 9.1 Log_2_ ED_50_/mL for the immune group and between 0.2 and 3.8 Log_2_ ED_50_/mL for the non-immune group. These titers did not change until the challenge.

After experimental infection with BRSV at day 0 (D0), the neutralizing antibody response increased in the non-immune group between D5 and D10 and reached similar levels to the immune group by the end of the experiment (D16). Conversely, there was no increase in the BRSV neutralizing antibody response in the immune group, suggesting that the maternal antibodies interfered with the development of the humoral response after infection.

### 3.2. Colostrum from the BRSV-Vaccinated Cows Protect Calves against Severe Disease after the Challenge

After the challenge, all non-immune calves showed clinical signs of respiratory tract infection that were moderate in five calves (38.4%), and severe in seven calves (53.8%). The two remaining non-immune calves only showed slight hyperthermia, with mucous nasal discharge and an infrequent cough. Moderate cases were characterized by hyperthermia, mucous nasal discharge, cough, increased respiratory rates (between 35 and 65 breaths/minute), and mild dyspnea, with increasing lung sounds but without consequences to their appetite or general state. Severe cases were characterized by highly increased respiratory rates (>65 cycles/minute), dyspnea including enhanced lung sounds with wheezes and crackles, a transient loss of appetite over 1–2 days, and slight depression. However, the clinical criteria for euthanasia according to the ethical protocol had not been met; we did not observe severe respiratory distress syndrome and all calves recovered. Variability within the immune group was also observed; five calves remained healthy, eight calves developed a mild form of BRD, and one calf showed moderate to severe clinical respiratory signs.

**Figure 2:**
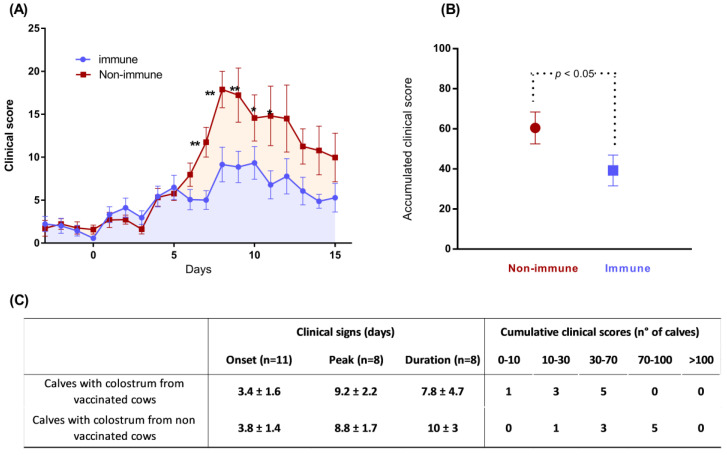
(**A**) Mean clinical scores with the standard errors of the mean (sem) (all calves), (**B**) mean accumulated clinical scores with sem (D0 to D16), (**C**) onset, peak, and duration of clinical signs and the number of calves with a cumulative clinical score of 0–10, 10–30, 30–70, 70–100 and >100 in groups receiving colostrum from Bovilis Bovigrip^®^-vaccinated cows (immune group) or from non-vaccinated cows (non-immune group). * = *p* < 0.05; ** = *p* < 0.01.

Statistical analyses indicated the group and time effects between the two groups for CS. Significant differences (Bonferroni’s test for contrast, D7 to D10: *p* < 0.01; D11: *p* < 0.05) were observed for the mean CS from D7 to D11, for RR at D7 and D8, and for the ACS (*p* > 0.05; Figure 2A,B). The onset, peak, and duration of the clinical scores were calculated for each group (Figure 2C), showing a shorter duration of clinical signs in the immune group, but with no significant differences (non-parametric Mann–Whitney U test, *p*-value one-tailed, *p* = 0.24). In addition, CCS showed a higher number of non-immune calves with high scores, suggesting that maternal antibodies protect against the severe clinical manifestation of the disease.

**Figure 3:**
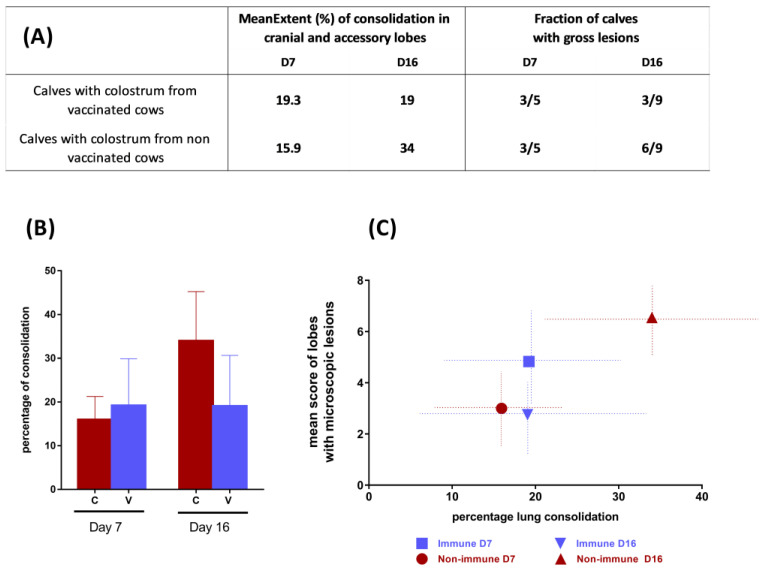
(**A**) Fraction of calves with gross lesions and extension of lung consolidation in the cranial and accessory lobes, between non-immune and immune groups at D7 and D16. (**B**) Mean histopathological score (+/−sd) of non-immune and immune groups (scores were established for each calf by the sum of scores of the cranial left, cranial right, and intermediate lobes). (**C**) Correlation between the total cranio-ventral percentage of lung consolidation (gross lesions, *x*-axis) and the mean scores of the microscopic lesions (*y*-axis).

At necropsy, the gross lesions of interstitial bronchopneumonia were restricted to the cranial and accessory lobes and were characterized by moderate consolidation and the presence of patchy atelectatic and collapsed areas, which were deep red and rubbery in texture. The extent of the macroscopic lesions was recorded for the cranial and accessory lobes and was shown in Figure 3A. To summarize, the extension of the consolidation varied between 5% and 70%, depending on the calf and the day of euthanasia. At D7, three out of five calves in each of the two groups showed macroscopic lesions. At D16, three and six calves (out of nine) in the immune and non-immune groups had gross lesions, respectively (Figure 3). The mean extension at D7 was 16% and 19% for the non-immune and immune groups at D7, and 34% and 19% at D16, respectively (Figure 3A).

Upon examination of the microscopic lesions, four out of the five calves of each group showed histopathological lesions at D7, while the situation was different at D16, with histopathological lesions found in eight and four calves of the non-immune and immune groups (*n* = 9), respectively. Five of the nine immune calves were free of lesions at D16. The inflammatory pattern at D7 correlated with the gross lesions and varied between very mild, nonspecific inflammatory changes and severe broncho-interstitial acute pneumonia with necrotizing bronchiolitis, the formation of bronchiolar epithelial syncytia, proliferative alveolitis with neutrophils and macrophages, and the infiltration of the alveolar septa by mononuclear cells (mainly lymphocytes). Lesions were observed in the tissue sections of the cranial and intermediate lobes. At D16, similar lesions were found in three calves, while others showed lesions representing subacute infection, characterized by hyperplasia of the bronchial epithelia, the proliferation of type II pneumocytes, the lymphoplasmacytic infiltration of alveoli and bronchi, and macrophagic alveolitis. The individual severity of the inflammatory responses was calculated in consolidated areas as the mean score of sections in the three sampled areas per calf (cranial lobes and the intermediate lobe). The severity scores were similar at D7 between the non-immune and immune groups (3 and 4.7 respectively, with no significant differences, Figure 3B). At D16, the differences were larger, with respective mean scores of 6.7 and 2.5 in the non-immune and immune groups, but with no significant differences, mainly due to high individual variability. In the immune group, only four calves showed histopathological lesions but had scores ranging between three (intermediate) and four (marked). Taken together, these results showed no significant differences in histopathology at D7, while the calves from vaccinated cows tended to have less extensive histopathological lesions at 16 days after the challenge. 

### 3.3. Colostrum from BRSV-Vaccinated Cows Does Not Protect Calves against BRSV Infection of the URT but Reduces the Viral Load in the Lung

**Figure 4:**
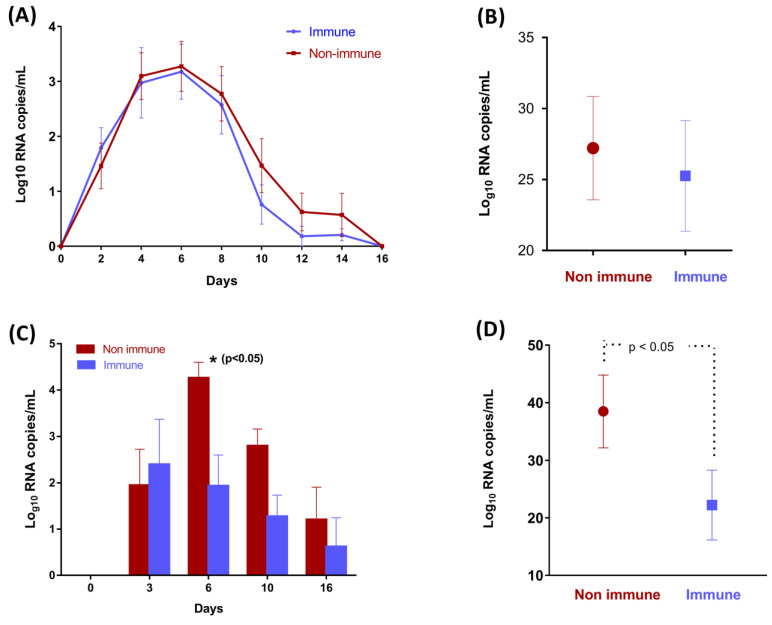
Mean BRSV daily RNA loads (all calves included) and mean accumulated BRSV shedding (8 calves per group) in the nasal secretions (**A**,**B**) and bronchoalveolar lavages (**C**,**D**), as determined by RT-qPCR (Log_10_ RNA copies/mL). Differences between the vaccine and control groups were not statistically different, except for BAL at D6.

No virus was detected via RT-qPCR in NS and BALs before the challenge in any of the calves. BRSV RNA was detected in the NS of all infected calves (Figure 4), with a peak level of virus shedding at D6 post-challenge (3.2 ± 0.5 and 3.3 ± 0.4 log10 of RNA copies for immune and non-immune groups, respectively). No significant differences were found between the two groups for BRSV RNA quantities in the nasal secretions or for accumulated virus shedding. The duration of the excretion was 7.9 ± 2.1 and 9 ± 2 days for the immune and non-immune groups. In the BALs, BRSV RNA was detected in five calves per group at D3, D6, D10, and D16 (Figure 4). BRSV was detected in larger quantities in non-immune calves at D6, D10, and D15 post-challenge, with statistical differences only for D6 (*p* < 0.038). A difference in the BRSV kinetic value was also observed in BALs, with a peak of replication at D3 for the immune group and at D6 for the non-immune group.

**Figure 5:**
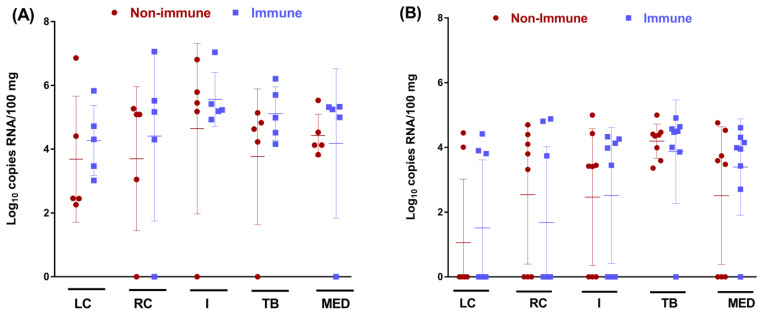
Individual BRSV loads (Log_10_ RNA copies/100 mg) with mean and SD, in the left cranial (LC), right cranial (RC), and intermediate (I) lung lobes, and in the tracheobronchial (TB) and mediastinal (MED) lymph nodes, in non-immune and immune calves at D7 (**A**) and D16 (**B**).

In addition, BRSV detection was also confirmed in the cranial, caudal, and intermediate lobes of the lung, as well as in the tracheobronchial and mediastinal lymphatic nodes of calves euthanized at D7 and D16. All calves were BRSV-positive for at least one tissue (Figure 5). BRSV RNA was detected in larger quantities at D7 (range of mean RNA loads of between 3.7 and 5.5 Log10 RNA copies/100 mg of tissues) than at D16 (between 1.1 and 3.6 Log10 RNA copies/100 mg of tissues). No statistical differences were found between the two groups for any tissue tested.

### 3.4. BRSV Maternal Antibodies Do Not Modify the Transcriptomic Host Response in the Lungs after the BRSV Challenge

**Figure 6:**
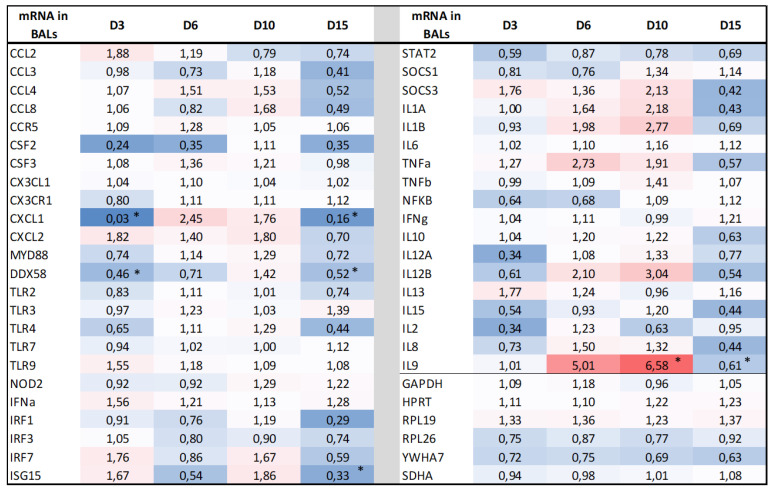
Comparative chemokine and cytokine mRNA expressions (assessed by an RT-qPCR Fluidigm assay) in BALs, expressed for each day, as the ratio of mean CNRQ values of immune over non-immune groups. The colors represent the method of modification of the ratio, from blue (ratio < 1) to red (ratio > 1), with coloring proportional to the values. Statistics were performed by comparing the slopes of the curves for each day (D3, D6, D10, or D15) on D0 between immune and non-immune calves, using a linear mixed model with a random effect for the group and considering the interactions between time and status. Statistical differences were indicated as follows *: *p* < 0.05.

Since BRSV was able to replicate in the LRT, we analyzed the BALs collected from pre-vaccinated and control calves for the transcriptomic response of 46 molecules. These included the pathogen recognition receptors (PRRs), cytokines, chemokines, and antiviral molecules involved in the interferon type I (IFN-I) response. Five time points were assessed: D0 was considered as a reference before the challenge, D3 corresponded to the initiation of IDV replication and innate immunity, D6 and D10 corresponded to the peak of the clinical signs, and D15 to the recovery of the inoculated calves. Four genes coding for STAT1, STAT3, IL4, and IL5 failed to pass the Fluidigm PCR quality check. Fold changes (the ratio of the mean CNRQ values of the vaccine to control groups after normalization on D0) and significant statistical differences between the two groups are shown in Figure 6. The log-transformed CNRQ data of the Fluidigm qPCR are available as supplementary Files S2 and S3. No significantly different expressions between the two groups were observed before infection.

Overall, the results showed similar levels of gene expression between the immune and the non-immune groups, with only a few significant differences when the statistics were recorded by comparing the slopes of the curves for each day (D3, D6, D10, or D15) on D0. Differences in expression were observed for some genes in the immune group, with a global overexpression at D6 and D10 and a downregulation at D15. Considering the IFN-I pathway, *RIG-1* was the only PRR (pattern recognition receptor) gene that was statistically under-expressed in the immune group at D3 and D15. Despite this, some genes involved in the interferon type-I signaling (*IFN-α*, *IRF7*, and *ISG15*) have fold change ratioslightly increased at D3 in the immune group but without statistical significance. Concerning the genes involved in inflammation, we did not find differences in mRNA expression of the CC and CXC classes of chemokines, except for the *CXCL1* gene, which was significantly under-expressed in the immune group at D3 and D15. On the other hand, the fold change ratio of this gene was increasedat D6 and D10, but without statistical significance. The upregulation of mRNA expression was also found at D5 and D10 for the proinflammatory interleukin genes, *IL1*, *IL6* and *TNFα*. The cellular adaptive immune response was also very similar between the two groups, except at D6 and D10 for the *IL12B* and *IL9* genes, and at D15 for *IL9* (significant for *IL9* at D10 and D15).

## 4. Discussion

One of the most widely accepted natural mechanisms of the disease protection of newborns against circulating microbes is maternal antibody transfer, which occurs in calves via colostrum administration shortly after birth. Passive immunization is indeed considered essential to prevent and fight infections in calves, such as diarrhea, respiratory disease, and/or septicemia, early in postnatal life. Although maternal antibodies were clearly shown to reduce the signs of acute BRSV infection during the first months of life [5,6,7,8,21,22,23,24,25], there are only a few experiments investigating the efficacy of vaccinating dams to protect the calves from BRD via maternal antibodies in the first weeks of life [24,29]. Makoschey et al. [29] have shown that colostral antibodies from cows vaccinated pre-partum with a trivalent killed vaccine against *Mannheimia haemolytica*, BPI3, and BRSV partially protect calves against the infection with *Mannheimia haemolytica*, as indicated by the higher level of *Mannheimia haemolytica*-specific antibodies in pre-vaccinated calves and the lower calf mortality rate when compared to the control group. Here, we showed that the vaccination of pregnant cows with the same vaccine before calving provides a significant reduction in clinical signs in calves challenged with BRSV at three weeks of age. All the calves fed with colostrum from non-vaccinated cows showed clinical signs, while 35.7% of the calves fed with colostrum from vaccinated cows were fully protected. We observed that the protective effect of pre-vaccination was mainly for severe clinical cases. Indeed, based on the clinical scores accumulated for each calf during the course of the experiment (Figure 2), it can be seen that only the non-immune calves (five out of nine) reached a CCS of above 70. This is also consistent with the individual daily clinical observations, where only these calves showed clinical signs of abdominal dyspnea, with moderate slaughter. Conversely, the CCS of the pre-vaccinated calves were in three ranges below 70 (Figure 2), and we did not observe any impairment of general condition for these calves. Assuming that severe clinical disease is linked to the infection of the LRT, this may correlate with the reduction in BRSV loads in the BALs of the immune group, starting at D6 post-challenge (Figure 4). In this group, the three calves that had the highest CS also had the highest BAL viral loads (between 10^3^ and 1.3·10^4^ RNA copies/mL). However, it was not possible to clearly confirm the correlations between CS and viral loads in the LRT of non-immune calves, possibly because only five calves per group were randomly selected for BALs. The more severe CS in the immune group is also consistent with more severe gross and microscopic lesions, at least at D16, although the differences are not statistically significant. However, we did not find significant differences between the two groups at D6 for lesions and for the BRSV RNA loads in the respiratory tissues. This is not consistent with the results of a previous study that examined the role of passive immunity in colostrum-deprived and colostrum-fed calves, using a similar model of dam vaccination and calf BRSV challenge [24]. These authors showed that colostrum, as the source of passive immunity, strongly reduced not only the severity of clinical signs but also the gross and microscopic lung lesions in colostrum-fed BRSV-inoculated calves. We do not know whether the differences are due to the early challenge of the calves, at days two to three of life in the previous study [24], versus 21 days in our study, at a time when the maternal antibody levels are at their highest. However, we did not find in our study that there was a significant decrease in SN antibody titers between birth and the day of the challenge (D0), suggesting a similar level of passive antibody protection during this period. Another explanation is that our BRSV challenge was more severe, as confirmed by the clinical and virological results in the non-immune group.

In the immune group, five calves still developed a mild clinical form of BRSV infection (range 3), although they had similarly high SN antibody titers, in comparison with other calves of the group at D0 and during the whole challenge period. We do not know why these calves had more severe clinical signs, despite having similarly high SN antibody titers. Further investigations are needed to answer this question, including the quality of the other components of the colostrum administered. Different factors may be involved in the individual variability of the calves, such as genetics or the health status of the animals, although we confirmed the absence of other respiratory pathogens and the seronegative status of the calves on the day of the challenge. In addition, a more detailed characterization of the nature of the neutralizing antibodies could be carried out. It was shown in the case of the human respiratory syncytial virus that the most potent epitopes for inducing neutralizing antibodies were shown to be conformation-dependent and unique to the pre-fusion F (pre-F) protein [30,31,32]. In addition, it was also shown that maternal pre-F antibodies are fundamental for providing immune protection to infants [33]. Consequently, one current view is that maternal immunization, which is considered one potential strategy to protect the neonate and infant in the first months of life, using vaccines containing the mutation-stabilized pre-F protein could be a safe and efficacious approach for the protection of infants against RSV [34,35,36]. Unfortunately, we do not know if the bovine-killed vaccine used in this study is able to induce pre-F antibodies. In addition, it is also well known that both the humoral and cellular responses are important against RSV infection. We did not directly study the cellular response and the potential interference by maternal antibodies in the calves after the challenge, but the transcriptomic assays showed no statistical differences in the *IL-2*, *IL-10*, *IL-12*, *IL-13* and *IFNγ* mRNA transcriptions in BALs. The only exception is for the *IL-9* mRNA, which is overexpressed at D6 and D10 and is downregulated at D15. In the mouse model of the RSV challenge, IL-9 was shown to regulate the pathology during the primary and memory responses to RSV infection [37]. In infants, high levels of IL-9 are present in the bronchial secretions of infants with RSV severe bronchiolitis [38,39], although an association of IL-9 with disease severity was not found [39,40]. The absence of the association is also observed in this study in calves, as an increased IL-9 mRNA expression was detected in the immune group at D6 and D10, at a time when clinical signs were comparatively less severe in this group. The other gene that was differentially expressed between the two groups is *CXCL1* at D3 and D15, which plays a role in inflammation and as a chemoattractant for neutrophils. Neutrophils were shown to be involved not only in the antiviral response against RSV disease but also in immunopathology when their recruitment and activation are not regulated [41,42]. Unfortunately, it was not possible to determine the cellular composition of BALs between the two groups in this study. In addition, the CCL-3 and CXCL-2 mRNAs, also encoding the protein chemotactic for polymorphonuclear leukocytes, were not differentially expressed between the two groups. Further studies are thus needed to determine why *CXCL1* is strongly repressed in the immune group so early after the challenge.

In recent years, a large number of studies have focused on the interference of maternal antibodies regarding the development of the humoral and cellular immune response after the vaccination of young calves, regardless of the type of vaccine or the route of immunization [5,8,10,11,12,13,15,17,18,19,20]. Some of these studies were able to reproduce severe clinical signs in the unvaccinated control group after the BRSV challenge, despite the presence of maternal antibodies [8,9,10,11,12,13]. The challenge conditions (including the virus strain, the dose, and the routes of inoculation), the health status of the calves, or the environmental conditions may explain these differences, as studies reproducing severe disease used a strain that was pre-amplified in calves and inoculated by nebulization [9,11,12,13,17,18]. In our study, using a similar pre-amplified strain but administered via intranasal and intratracheal routes, we were not able to induce the highly severe disease that would justify early end-point euthanasia. However, the clinical signs were sufficiently important in the non-immune group and corresponded to the vast majority of the clinical forms observed in the field, justifying veterinary intervention. This partly validates the challenge model used in this study. Another explanation is more likely the age of the calves and, consequently, the level of antibodies present at the challenge. In our study, we used optimal conditions for colostrum transfer, and we infected calves at 21 days of age, when their SN antibody titers were high (Table 1). In contrast, in vaccine interference studies, the challenge was usually carried out after 3 months, at an age when some of the maternal antibodies have disappeared or when the titers are moderate. This suggests that clinical protection is partly dependent on the level of SN antibodies present in the calf at the time of infection, and is, therefore, limited in time. The levels of BRSV maternal antibodies vary greatly in dairy calves fed colostrum from their own dams, from titers < 1:16 to >1:512 when tested within 2 days after birth [37]. Similar titers were obtained in studies on the interference between passive immunity and vaccination, which quantified the SN maternal antibody titers of calves. In one study, the level of BRSV SN antibody was standardized in the colostrum (colostral titer of 1:64) and the calves’ BRSV titers ranged from 1:16–1:64, with an average value of 1:32 at 36 h following feeding [15]. In two other studies where the calves received whole colostrum, the SN titers were at 1:16 when tested later after birth, at the date of vaccination [14,19]. In our study, at D21 of the challenge, the SN antibody titers of the immune group ranged between 7.1 and 9.2 Log2 EID_50_/mL, corresponding to titers of between 1/16 and 1/64. These titers are similar to those used by Kolb et al. [15] but, unfortunately, in the Kolbs study, non-immune calves were challenged when calf BRSV SN titers had declined to < 1:4. In field conditions, a positive correlation between the high levels of SN antibodies derived from colostrum and a decreased incidence of BRDC has been previously reported in calves [43,44,45].

The duration of passively acquired immunity, expressed as the mean time to reach seronegative status, is between 5.2 and 8 months [46,47]. However, the duration of the protective period afforded by the maternal antibodies could be related to several factors in the field, including the quantity and the nature of the antibodies produced by the dams after vaccination and, therefore, found in the colostrum, the quality of the colostrum transfer to the calves immediately after birth, and finally, the initial titer absorbed from the maternal colostrum [48,49]. These parameters could explain the large diversity of situations observed in the field and the heterogeneity of calves, in terms of their SN maternal antibody titers. The range of maternal antibody titers to respiratory viruses after colostrum intake is highly variable among calves [43,46,48]; its coefficient of variation in a group of two-day-old calves was 24.98% for BRSV [46].

Despite this clinical protection, the presence of maternal antibodies in calves from vaccinated dams does not prevent BRSV infections of the URT. We also did not find any differences in BRSV replication between the calves of both groups in relation to the individual level of the neutralizing antibodies and the afforded clinical protection. Although we did not measure antibodies in the nasal secretions, it can be assumed that since the maternal antibodies are mainly IgG1, they will not be present or at low levels in the upper respiratory tract. In the field, this suggests that the vaccination of pregnant cows with Bovilis Bovigrip vaccine probably has no effect on BRSV circulation among the calves. However, we used a challenge model with high infectious BRSV titers. Natural BRSV infection would be much milder than our challenge infection, resulting in greater efficacy of the vaccination to reduce virus excretion. We can also speculate that the active immunization of cows will reduce BRSV excretion after natural challenges, as indicated in the official product specifications. To date, we do not know the impact of dam vaccination on silent BRSV circulation in cattle herds.

## 5. Conclusions

This study indicates that the vaccination of pregnant cows with Bovilis Bovigrip before calving provides (i) a strong antibody response in cows with no or low levels of initial BRSV antibodies, (ii) the presence of high quantities of BRSV-neutralizing antibodies in the colostrum, and (iii) a significant reduction in the clinical signs in calves challenged with BRSV at 3 weeks of age. This may represent an alternative approach to protecting the calf early in life to achieve higher and more homogenous levels of antibodies in the colostrum and also of specific memory cells in the calves [10]. It is clear that our experience is in optimal conditions for the vaccination and transfer of maternal immunity, which is not always the case in field conditions. In addition, we do not know the duration of maternal antibody protection after 21 days of life. Vaccination of the dams, if used at all, must take all these factors into account and must be complementary to an overall disease control plan, including the improvement of maternal antibody transfer, biosecurity, and association with the vaccination of calves.

## Figures and Tables

**Table 1:** BRSV antibodies were tested via ELISA (Life Technologies—LsiVRSB ELISA kit, France) and seroneutralization (SN) in cows before and after vaccination, in the colostrum, and in calves at D-19 (two days after calving), at D0 just before the challenge, and at D15–D16 post-challenge. The indirect ELISA columns indicate the number of animals or colostrum samples that are negative (Neg), weakly (+), moderate (++), highly (+++) and very highly positive (++++), according to the manufacturer’s score. SN titers are expressed as the Log_2_ of ED_50_/mL (50% effective dose) according to the Spearman–Kärber titration in the range.

	Date	ELISA (Number of Animals)		Neutralization (Mean Titers with Range in Square Brackets; Log2 EID50/mL)	
		Non-Vaccinated	Vaccinated	Non-Vaccinated	Vaccinated
**Cows** (*n* = 16/group)	Before vaccination	Neg (6) + (6) ++ (4)	Neg (6) + (6) ++ (4)	2.1 [0–3.4]	2.4 [0–3.9]
	Two weeks after vaccination	Neg (6) + (6) ++ (4)	++ (1) +++ (2) ++++ (13)	2.3 [0–3.4]	8.6 [7–9.2]
**Colostrum** (*n* = 16/group)		Neg (12) + (4)	++ (4) +++ (4) ++++ (8)	1.8 [0–7.6]	9.4 [6.1–11.7]
**Calves** (ELISA, *n* = 14/group) (SN, *n* = 7/group)	D-19	Neg (6) + (4) ++ (4)	++ (4) +++ (5) ++++ (5)	0.9 [0.2–3.8]	8.8 [7.2–9.1]
	D0	Neg (6) + (4) ++ (4)	++ (4) +++ (5) ++++ (5)	0.6 [0–3.8]	8.4 [7.1–9]
	D16	++ (2) +++ (6) ++++ (6)	++ (3) +++ (6) ++++ (5)	8.7 [7.5–10.5]	8.8 [8.3–9.3]

## Data Availability

Not applicable.

## Supplementary Materials

The following supporting information can be downloaded at: https://www.mdpi.com/article/10.3390/vaccines11010141/s1, Supplementary File S1: List of primers used for the transcriptomic assays. Supplementary File S2: chemokine and cytokine mRNA expressions in BALs, expressed for each day, by the mean CNRQ values of immune over non-immune groups. The colors represent the way of modification of ratio, from blue (ratio < 1) to red (ratio > 1) with a coloring proportional to the values. Supplementary File S3: Raw data of CNRQ values of chemokine and cytokine mRNA expressions in BALs.
